# Amperometric Self-Referencing Ceramic Based Microelectrode Arrays for D-Serine Detection

**DOI:** 10.3390/bios8010020

**Published:** 2018-03-06

**Authors:** Diana Campos-Beltrán, Åsa Konradsson-Geuken, Jorge E. Quintero, Lisa Marshall

**Affiliations:** 1 Institute of Experimental and Clinical Pharmacology and Toxicology, University of Lübeck, Ratzeburger Allee 160, 23562 Lübeck, Germany; diana.campos@pharma.uni-luebeck.de; 2 The Department of Pharmaceutical Biosciences, Uppsala University, 75124 Uppsala, Sweden; asa.konradsson-geuken@farmbio.uu.se; 3 The Department of Physiology and Pharmacology, Karolinska Institutet, 17177 Stockholm, Sweden; 4 CenMeT, University of Kentucky, Lexington, 40506 KY, USA; george@quanteon.cc; 5 Quanteon LLC, Nicholasville, 40356 KY, USA

**Keywords:** D-serine, biosensor, microelectrode array, amperometry, self-referencing

## Abstract

D-serine is the major D-amino acid in the mammalian central nervous system. As the dominant co-agonist of the endogenous synaptic NMDA receptor, D-serine plays a role in synaptic plasticity, learning, and memory. Alterations in D-serine are linked to neuropsychiatric disorders including schizophrenia. Thus, it is of increasing interest to monitor the concentration of D-serine in vivo as a relevant player in dynamic neuron-glia network activity. Here we present a procedure for amperometric detection of D-serine with self-referencing ceramic-based microelectrode arrays (MEAs) coated with D-amino acid oxidase from the yeast *Rhodotorula* *gracilis* (RgDAAO). We demonstrate in vitro D-serine recordings with a mean sensitivity of 8.61 ± 0.83 pA/µM to D-serine, a limit of detection (LOD) of 0.17 ± 0.01 µM, and a selectivity ratio of 80:1 or greater for D-serine over ascorbic acid (mean ± SEM; *n* = 12) that can be used for freely moving studies.

## 1. Introduction

In the 1990’s, the occurrence of D-serine in the mammalian brain and the similarity in its distribution pattern to that of the N-methyl D-aspartate receptor (NMDAR) were first reported [1,2]. D-serine has since been confirmed as the main endogenous co-agonist of the glycine modulatory site of the NMDAR in corticolimbic areas of the brain [3,4,5]. As such, it is presumed to be crucially involved in neuroplasticity and cognitive functions, in particular, for learning and memory [5,6,7,8,9,10,11,12,13,14,15,16,17,18]. Furthermore, D-serine has been attributed a role in the hyper- and/or hypofunction of the NMDAR in neuropsychiatric disorders [19,20,21]. For example, a major component in the pathophysiology of schizophrenia, in particular, the cognitive deficits, is suggested to be NMDAR hypofunction associated with reduced D-serine levels [12,20]. In the brain, D-serine is synthesized in neurons and/or astrocytes from L-serine by the enzyme serine racemase [22]. Endogenous degradation after cellular uptake can occur via the enzyme D-amino-acid oxidase (DAAO), located in peroxisomes, although for the brain other mechanisms are also discussed [23].

Thus, independent of the ongoing debate on the neuronal and/or glial sources of D-serine [22,24,25,26,27], its level and dynamics are pressing issues for understanding cognitive impairment and neuropsychiatric diseases, as well as for basic research in neuroplasticity.

While several types of biosensors have already been employed for amperometric D-serine detection [18,28,29,30,31,32,33,34], the extracellular levels of D-serine in the brain of freely moving rodents have so far only been measured by microdialysis [32,35,36,37,38]. A main advantage of amperometric recordings is the ability to detect neurochemical changes in the range of seconds [39,40,41]. Such high temporal resolution is relevant not only to detect putative fast transients as found for glutamate [41], but also for monitoring neurochemical changes in relation to both state-dependent [42], as well as fast changes in brain electric activity. Here, we provide a protocol for amperometric D-serine measurement with D-amino acid oxidase (DAAO) coated ceramic-based microelectrode arrays (MEAs), whose configuration allows for offline self-referencing. The principle of self-referencing employs 2 types of channels, here with each consisting of a pair of platinum recording sites: D-serine detecting channels and sentinel channels. Both of the channel types are able to measure background noise and interferent activity, but only the enzyme coated sites (D-serine detecting channels) are able to detect D-serine [43,44,45]. Background noise and neurochemical interferent activity can thus be easily subtracted from the D-serine detecting channels [43,44,45]. Sentinel and D-serine detecting channels are spaced only tens of micrometers apart on the MEA [44]. In fact, dual-sided MEAs with eight recording sites have been used to record simultaneously in multiple regions of the prefrontal cortex [46]. With specific configurations, recordings of different analytes with one MEA [47,48] and of local field potentials in parallel using a high data acquisition rate were conducted [48,49,50]. MEAs possess a high spatial resolution, and are made of a biocompatible material having been shown to produce only minimal tissue damage [44,51], a feature that is required for chronic recordings.

## 2. Materials and Methods

### 2.1. Chemicals

L-ascorbic acid (AA), D-serine, D-alanine, L-glutamate, dopamine hydrochloride (DA), bovine serum albumin (BSA), sodium chloride (NaCl), sodium phosphate monobasic monohydrate (NaH_2_PO_4_), sodium phosphate dibasic anhydrous (Na_2_HPO_4_), *meta*-phenylenediamine dihydrochloride (*m*PD), and glutaraldehyde were purchased from Sigma-Aldrich (St. Louis, MO, USA). Hydrogen peroxide (H_2_O_2_) 3% (Paul W. Beyvers GmbH, Berlin, Germany) was purchased at a local pharmacy. The recombinant D-amino acid oxidase from the yeast Rhodotorula gracilis (RgDAAO, EC 1.4.3.3) was purified, as stated in Fantinato et al., [52], and was purchased with an approximate activity of D-serine given as 20 U/mg dry protein (and 100 U/mg dry protein on D-alanine) from “The Protein Factory research center” (Milano, Italy). Porcine kidney D-amino acid oxidase (pkDAAO, EC 1.4.3.3) with a specific activity of D-alanine given as 2.53 U/mg dry weight protein (data on D-serine was not provided) was obtained from Worthington Biochemical Corporation (Lakewood, NJ, USA). Stock solutions were prepared using deionized distilled water, except in the case of dopamine that was mixed with 1% perchloric acid (PCA) in addition, for a long shelf life. The 0.05 M phosphate-buffered saline (PBS) solution used for in vitro calibration had a pH close to 7.4, mimicking the physiological levels. A 5 mM *m*PD solution was made using nitrogen saturated PBS to reduce the oxidation of the compound.

### 2.2. MEA Preparation

**Figure 1:**
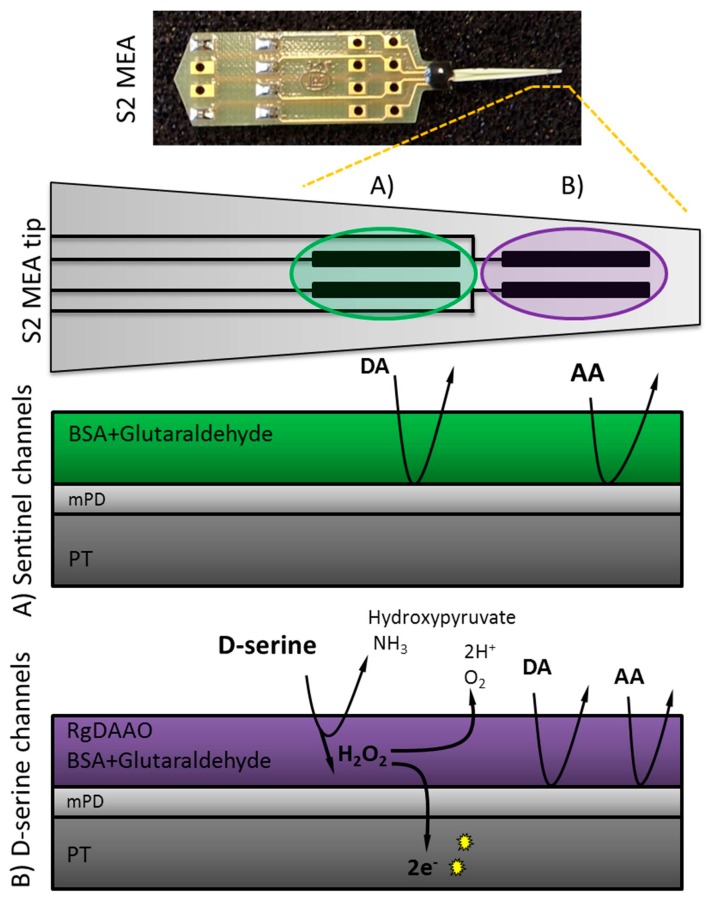
Scheme for D-serine detection using S2 style Microelectrode Array (MEAs) consisting of two pairs of platinum (PT) sites. When D-serine comes into contact with *Rhodotorula gracilis* (RgDAAO) (platinum sites **B**: D-serine recording channels) H_2_O_2_ is produced by the enzymatic reaction, crosses the *m*PD barrier and is then further oxidized, yielding two electrons per molecule. At the amperometric fixed potential of +0.7 V, the sentinel channels (platinum sites **A**) detect only electrochemically active interferents and background noise, whereas the RgDAAO coated channels measure in addition D-serine.

Ceramic-based S2 MEAs were obtained from CenMeT (Lexington, KT, USA; a comprehensive fabrication review can be found elsewhere [44]). The S2 style MEA is arranged as two pairs of platinum recording sites of 333 × 15 µm, separated by 30 µm from the neighboring recording site and by 100 µm from the other pair (cp. Figure 1) [44]. After arrival, MEAs were cleaned and calibrated in vitro using H_2_O_2_ to test performance (data not shown). Subsequently, MEAs were enzyme coated (see “Enzyme preparation and immobilization” section) and stored at −20 °C until use, to maintain enzymatic stability. An exclusion layer was applied (see “Electropolymerization of *meta*-phenylenediamine (*m*PD)”) one day prior to calibration (see “In vitro calibration and recording parameters”). In-depth background on the relevance of these steps has been reported elsewhere in regard to other enzyme-based MEAs [43,44,53].

For the freely moving recording the printed circuit board of the MEA was soldered onto a miniature Omnetics connector (Omnetics Connector Corporation, Minneapolis, MN, USA) and a chloride coated silver (Ag/AgCl) wire was used as reference electrode (Teflon-coated silver wire, 125 µm bare diameter, A-M Systems, Inc., Sequim, WA, USA). This MEA configuration enables the use of a smaller preamplifier and thus, reduces weight of the headstage on the rat.

### 2.3. Enzyme Preparation and Immobilization

The MEA configuration allows for offline self-referencing: two enzyme-coated platinum sites were used for detecting D-serine (in Figure 1B), while two sentinel channels (in Figure 1A) detected other potential electroactive compounds that could likewise be oxidized at +0.7 V, e.g., endogenous H_2_O_2_. Measurements recorded with the sentinel channels are subtracted offline from the D-serine detecting channels to obtain an interference-free D-serine signal. For coating, one droplet from an aliquot of each 1% BSA, 0.125% glutaraldehyde, and 0.1 U/µL RgDAAO solution was applied to the two D-serine recording channels under a stereomicroscope (0.8×–4×; Olympus Optical Company, Hamburg, Germany) using a Hamilton microsyringe (Hamilton Company, Reno, NV, USA). Similarly, a chemically inactive protein matrix composed of only BSA and glutaraldehyde was cross-linked onto the sentinel channels. Following the same procedures as described above, some MEAs (*n* = 4) were coated with mammalian DAAO purified from porcine kidney.

### 2.4. Electropolymerization of Meta-Phenylenediamine (mPD)

*Meta*-phenylenediamine (*m*PD) served as an exclusion layer to increase the selectivity for D-serine. The layer of *m*PD functions as a size exclusion barrier, prohibiting electroactive substances, such as AA or DA, from reaching the platinum sites of the MEA [44]. *m*PD was electropolymerized onto all platinum sites (D-serine recording and sentinel channels) after coating using a cycled potential between +0.25 and +0.75 V vs. Ag/AgCl at a frequency of 0.05 V/s in a 5 mM *m*PD solution for 22 min (FAST electroplating tool, Quanteon, LLC, Lexington, KT, USA). Note, *m*PD electropolymerization occurs onto the surface of the platinum sites of the MEA, below the enzyme layer [44].

### 2.5. In Vitro Calibration and Recording Parameters

Enzyme coated MEAs were calibrated in vitro to determine their sensitivity to D-Serine (pA/µM D-serine), selectivity against ascorbic acid or glutamate, limit of detection (LOD), and linearity (R^2^) under an amperometric fixed potential of +0.7 V versus a glass Ag/AgCl reference electrode (RE-5B Ag/AgCl, ProSense, Oosterhout, The Netherlands). Signals were first preamplified using a microamplifier (2 pA/mV 500×; Quanteon, LLC, Lexington, KT, USA) and then amplified and digitized at 10 Hz with the FAST-16mkIII data acquisition system (Quanteon, LLC, Lexington, KT, USA). Standard calibrations were performed by immersing the MEAs in a calibration media, which consisted of phosphate buffered-saline (PBS, 40 mL). The solution was stirred using a magnetic stirrer device (Stuart, Bibby Scientific Limited, Staffordshire, UK) and kept at body temperature in a water bath chamber (Pronexus, Analytical AB, Stockholm, Sweden) at 37 °C controlled by a water heater system (Micro-Temp LT, Cincinnati Sub Zero, Cincinnati, OH, USA).

According to a standard protocol [43,44,45,53,54,55], after a stabilization period of at least 30 min, aliquots of AA, D-serine, DA, and H_2_O_2_ were given. For the D-serine calibration three consecutive 40 µL additions of a 20 mM D-serine solution produced three consecutive total concentrations in the beaker of 20 µM, 40 µM, and 60 µM. For AA the resulting concentration was 250 µM (500 µL of 20 mM), for DA 2 µM (40 µL of 2 mM), and for H_2_O_2_ 8.8 µM (40 µL of 8.8 mM); *n* = 12 for RgDAAO coated and *n* = 4 for pkDAAO coated MEAs.

**Figure 2:**
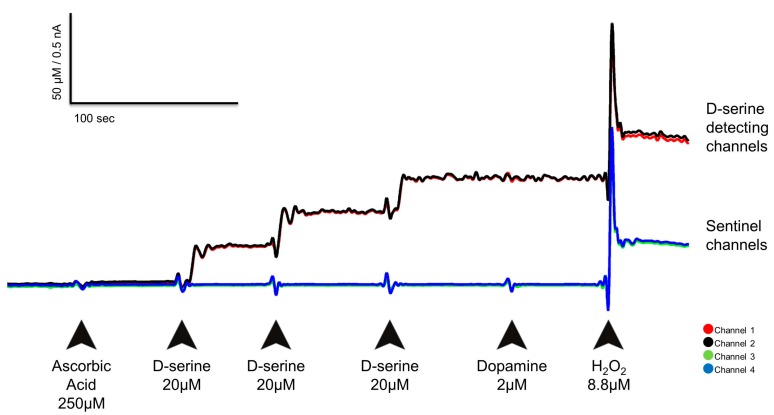
In vitro calibration of one D-serine detecting Microelectrode Array (MEA). Arrows indicate when substances were added and the resultant concentration obtained in the calibration media. The figure reflects the current (nA) and corresponding concentration (µM) measured by the MEA after each challenge. Channels 1 and 2 are D-serine detecting, and channels 3 and 4 are sentinel channels, respectively. See Table S2 and Figure S2 for parameters of this calibration, and Figure S1 and Table S1 for the calibration using 1 mM D-serine.

We selected these concentrations as they belong to an established in vitro calibration methodology using enzyme-based MEAs for the detection of other molecules like L-glutamate [43,44,45,53,54,55,56]. Thus, our calibration results are directly comparable to previous calibration measurements, although a much lower basal D-serine concentration in the prefrontal cortex of the anesthetized rat was reported (~3 µM) [28]. Moreover, calibrations using only 1 mM D-serine (reaching a final concentration in the calibration media of 1 µM) met the same threshold criteria (*n* = 5) as the calibrations using a 20 mM D-serine solution (Figure S1 and Table S1). Figure 2 depicts a typical calibration of a MEA from which the following parameters were derived: selectivity for D-serine against AA (concentration ratio of D-serine over AA), sensitivity to D-serine (pA/µM D-serine), limit of detection (LOD) for D-serine (in µM), and linearity (R^2^; FAST analysis 6.1 software; Jason Burmeister Consulting, LLC, KT, USA). Hereby, the responsivity of the MEA to changes in concentrations of D-serine is indicated by the sensitivity; the LOD (i.e., the lowest change in D-serine detected by the DAAO coated MEAs that cannot be attributed to noise, calculated as three times the standard deviation of the noise of the corresponding MEA channel during calibration), and the linear regression shows the linear response to the accumulating concentrations of D-serine that were added during the calibration [44,45]. Hydrogen peroxide was added to check for equal response performance to the reporter molecule of all the channels (data not shown) [42].

Two other calibrations of the RgDAAO coated MEAs were performed to test selectivity against L-glutamate and sensitivity to D-alanine. For the former, a final concentration of 20 µM of L-glutamate instead of AA were added to the calibration media (*n* = 10). D-alanine is a substrate for DAAO as well, thus the sensitivity to D-alanine was tested adding three aliquots of D-alanine to create a final concentration of 20 µM, 40 µM, and 60 µM in the calibration media (*n* = 12). All data are presented using mean ± standard error of the mean (SEM).

### 2.6. Surgery and Freely Moving Test of the D-Serine MEAs

As proof of principle, a RgDAAO coated MEA was implanted in vivo in a male Long Evans rat (15 weeks at time of surgery; Janvier, Le Genest-Saint-Isle, France). All of the experimental procedures were performed in accordance with the European animal protection laws and policies (directive 86/609, 1986, European Community) and were approved by the Schleswig-Holstein state authority. The animal was housed individually with ad libitum access to food and water under a 12 h/12 h light-dark cycle (lights on at 06:00 A.M.), and was handled seven days prior to surgery.

Stereotaxic surgery took place under isoflurane anesthesia. The MEA was implanted in the prefrontal cortex (AP: +2.5 mm, L: −0.5 mm, DV: 2.5 mm) using an anterior Ag/AgCl electrode as reference (AP: +5.5 mm, L: +1.0 mm, DV: 2.5 mm) [57]. The prefrontal cortex was selected due to its reported high levels of D-serine [3,35].

After seven days of recovery, the freely moving recording was performed in a dark PVC recording box with the same amperometric system as described for calibrations using cabling attached to a low torque slip-ring commutator (Dragonfly Research and Development, Inc., Ridgeley, WV, USA). D-serine signals were obtained after a one hour baseline recording during which channels stabilized.

Analyses were conducted with the FAST analysis 6.1 software as mentioned above. Only transients measured in the D-serine channels and not in the sentinel channels were included in the analyses.

## 3. Results and Discussion

### 3.1. In Vitro Calibration Results

Mean values for the calibration parameters are given in Table 1, together with the threshold criteria for sufficient MEA properties [44,45]. Our RgDAAO coated MEAs demonstrated a sensitivity of 8.61 ± 0.83 pA/µM to D-serine, a linearity of 0.9986 ± 0.0005 and an LOD of 0.17 ± 0.01 µM (mean ± SEM; *n* = 12, using a data acquisition rate of 10 Hz. In addition, all of the channels on a given MEA revealed a similar response to H_2_O_2_; meaning that D-serine recording and sentinel channels only differed in their response to D-serine (data not shown) [42].

### 3.2. Selectivity

In addition to the use of enzymes, selective detection of non-electroactive molecules, such as D-serine, is achieved by using size exclusion layers which form a barrier for larger interfering electroactive molecules (e.g., ascorbic acid). While in the past Nafion^®^ was utilized as an exclusion layer [44,56], Meta-phenylenediamine (*m*PD) has proven to be a better barrier against interfering molecules as monoamines are attracted by Nafion^®^ [45,46,58,59]. After electropolymerization of *m*PD onto the MEA only small molecules like H_2_O_2_ can readily cross this layer and reach the platinum sites. We tested our D-serine MEAs against AA and L-glutamate (Table 1) since both of them are found at high concentrations in the same brain regions as D-serine, and glutamate is involved in the release of both AA and D-serine [8,60,61,62,63]. Glutamate should not be detected by the D-serine-selective MEAs as a different enzyme (glutamate oxidase) is required to transform glutamate into a measurable compound, but AA is electroactive and therefore a possible interferent. Selectivity is the ratio of the sensitivity for D-serine over the interferent and has to fulfill our criteria of being higher than 80:1. This means an increase in interferent concentration above 80 µM would be necessary to produce a signal corresponding to a 1 µM increase of D-serine [44]. Our RgDAAO coated MEAs effectively blocked both AA and glutamate, with a mean selectivity for D-serine against AA and L-glutamate of 191.75 ± 19.55 and 521.3 ± 112.23, respectively (*n* = 12 and *n* = 10; mean ± SEM; Table 1).

### 3.3. Other D-Amino Acids

D-amino acids aside from D-serine can also be a substrate of D-amino acid oxidase. D-aspartate, which is the second most abundant D-amino acid, is not detected by D-serine biosensors because it is not a substrate of DAAO, but D-alanine, the third most abundant D-isomer found in the mammalian brain, can be detected (e.g., RgDAAO MEAs had a sensitivity of 18.56 ± 1.6 pA/µM to D-alanine; *n* = 12). Yet, the endogenous concentration of D-alanine in the brain is only 3% of the D-serine concentration and can be thus considered negligible for in vivo measurements [28,64].

### 3.4. RgDAAO vs. pkDAAO

The choice of DAAO from a yeast, *Rhodotorula gracilis* (RgDAAO), over the mammalian DAAO from porcine kidney (pkDAAO) was made given the previously reported superior enzymatic properties of RgDAAO [65,66]. Other studies have demonstrated a tolerable sensitivity using pkDAAO [28], but the parameters that were obtained from our first calibrations using pkDAAO did not fulfill our criteria: the sensitivity of the pkDAAO coated MEAs to D-serine was 0.45 ± 0.2 pA/µM and the LOD was 2.93 ± 0.7 µM (*n* = 4). Since the enzyme layers, BSA and glutaraldehyde layers were inspected under a stereoscope (0.8×–4×; Olympus Deutschland GmbH. Hamburg, Germany), as for all calibrated MEAs, the poor parameters obtained with pkDAAO cannot be attributed to peeling, cracking, or swelling of the coatings, the insulation layer or the platinum sites per se. Consequently, we employed D-amino acid oxidase from the yeast Rhodotorula gracilis for all further measurements.

### 3.5. Freely Moving Measurement of D-Serine

**Figure 3:**
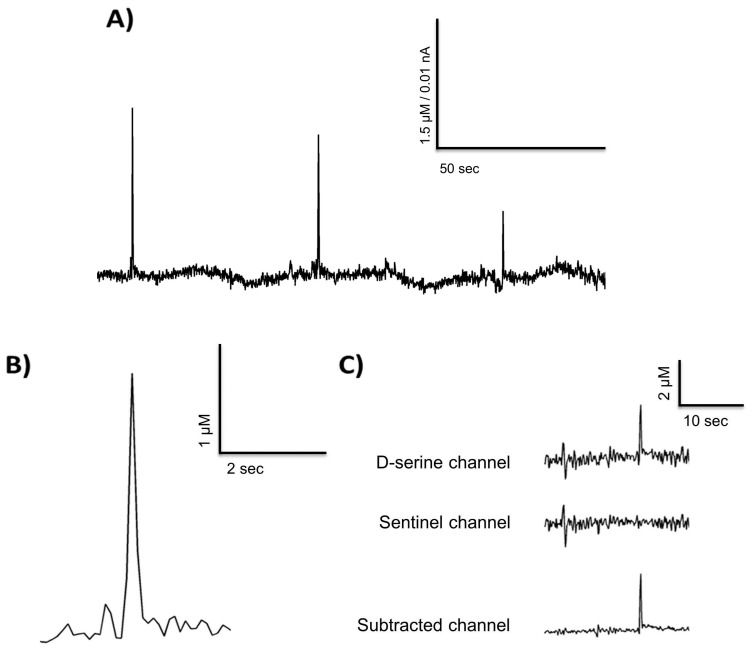
D-serine transients from a freely moving recording in the rat prefrontal cortex (**A**,**B**) and a representative example of the self-referencing method used (**C**).

Figure 3A depicts spontaneous D-serine transients recorded from the prefrontal cortex in a freely moving animal. A close up of one transient is depicted in Figure 3B. These exemplary measurements in a freely moving animal suggest that D-serine could be released into the extracellular space in fast transients as has been reported for glutamate [41,43,44,45,56]. A number of 89 D-serine transients were found in a 10 h recording, with a mean frequency of five per hour. Thus, we demonstrate that RgDAAO coated MEAs can be used to record in vivo, but further measurements have to be performed on a larger sample of animals to characterize any changes in basal D-serine levels and in the parameters of the transients (e.g., amplitude, frequency, temporal occurrence) and relate them to behavioral states [32,42] of freely moving animals.

## 4. Conclusions

We demonstrate here a reliable in vitro protocol for preparing RgDAAO coated multisite MEAs for fast and selective amperometric measurements of D-serine. Our preliminary results in the freely moving recording support the need for a recording technique with a high temporal resolution to detect fast D-serine dynamics in the mammalian brain. Further in vivo studies using freely moving animals need to be conducted given the recent findings on the functions of D-serine within the central nervous system.

## Figures and Tables

**Table 1:** In vitro measurements for the RgDAAO coated D-serine detecting MEAs as compared to threshold criteria (*n* = 12; mean ± SEM). To test for selectivity against L-Glutamate 10 calibrations were performed.

Parameters	Sensitivity	LOD	Linearity (R^2^)	Selectivity Against AA	Selectivity Against L-Glutamate
Threshold criteria	≥2 pA/µM	≤0.5 µM	1	≥80	≥80
Measurements	8.61 ± 0.83 pA/µM	0.17 ± 0.01 µM	0.9986 ± 0.0005	191.75 ± 19.55	521.3 ± 112.23

## Supplementary Materials

The following are available online at http://www.mdpi.com/2079-6374/8/1/20/s1, Figure S1: In vitro calibration of one D-serine detecting Microelectrode Array (MEA) using 1 mM D-serine challenges, Figure S2: Linearity (R^2^) curve obtained from the calibration of the RgDAAO coated D-serine detecting MEA depicted in Figure 2, Table S1: In vitro calibration measurements for the RgDAAO coated D-serine detecting MEAs using final concentrations of 1 µM D-serine in the solution media (*n* = 5; mean ± SEM), Table S2: In vitro calibration measurements obtained from the calibration of the RgDAAO coated D-serine detecting MEA depicted in Figure 2.
