# Supplementary material for: Amperometric Self-Referencing Ceramic Based Microelectrode Arrays for D-Serine Detection
Source: Biosensors (Basel). 2018 Mar 6;8(1):20. doi: 10.3390/bios8010020 (PMC5872068; doi:10.3390/bios8010020)
Supplement: Supplementary file 1 [file biosensors-08-00020-s001.pdf]

biosensors

MDPI

Supplementary Materials: Amperometric Self-Referencing Ceramic Based Microelectrode Arrays for D-Serine Detection

Diana Campos-Beltrán [1], Åsa Konradsson-Geuken [2,3], Jorge E. Quintero [4,5] and Lisa Marshall [1,*]

[1] Institute of Experimental and Clinical Pharmacology and Toxicology, University of Lübeck, Ratzeburger Allee 160, 23562 Lübeck, Germany; diana.campos@pharma.uni-luebeck.de
[2] The Department of Pharmaceutical Biosciences, Uppsala University, 75124 Uppsala, Sweden; asa.konradsson-geuken@farmbio.uu.se
[3] The Department of Physiology and Pharmacology, Karolinska Institutet, 17177 Stockholm, Sweden
[4] CenMeT, University of Kentucky, Lexington, 40506 KY, USA; george@quanteon.cc
[5] Quanteon LLC, Nicholasville, 40356 KY, USA
* Correspondence: lisa.marshall@pharma.uni-luebeck.de; Tel.: +49-451-3101-7223; Fax: +49-451-3101-7204

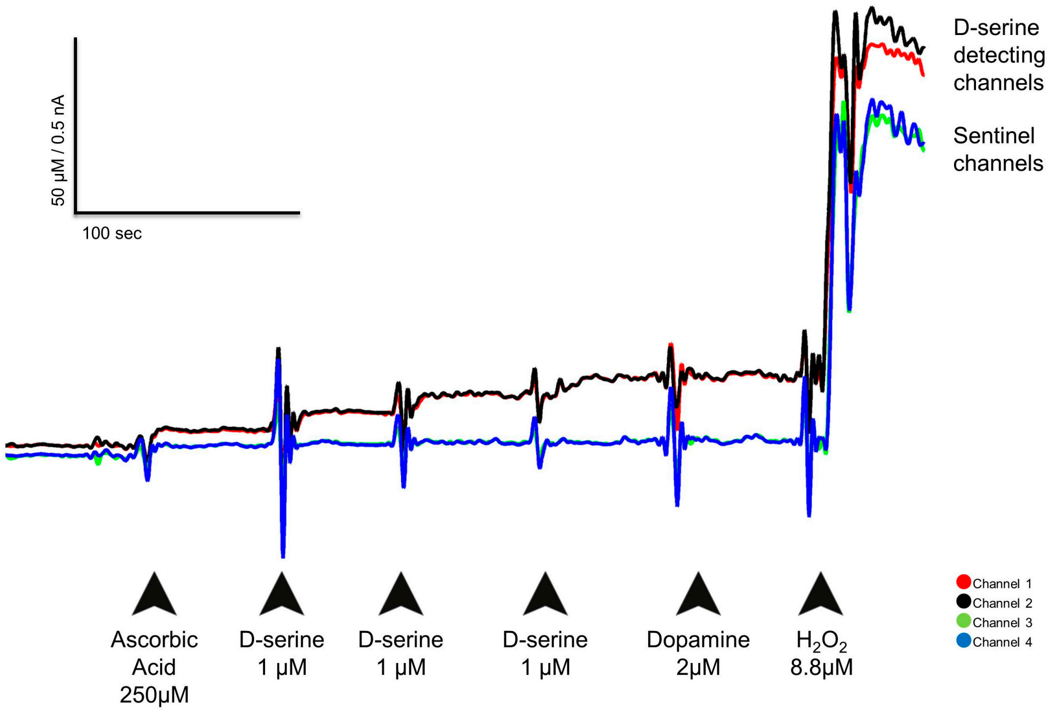

Figure S1. In vitro calibration of one D-serine detecting Microelectrode Array (MEA) using 1 mM D-serine challenges. Arrows indicate when substances were added and the resultant concentration obtained in the calibration media. The figure reflects the current (nA) and corresponding concentration (µM) measured by the MEA after each challenge. Channels 1 and 2 are D-serine detecting, and channels 3 and 4 are sentinel channels, respectively. Note that the higher resolution of the picture leads to a higher visibility of background noise.

Table S1. In vitro calibration measurements for the RgDAAO coated D-serine detecting MEAs using final concentrations of 1 μM D-serine in the solution media (n=5; mean ± SEM). There was a minimal decrease in linearity as compared to the standard calibration and the limit of detection (LOD) increased slightly.

**Parameters**	**Sensitivity**	**LOD**	**Linearity (R^2)**
1 μM challenge calibration measurements	8.67 ± 0.0018 pA/μM	0.25 ± 0.06 μM	0.9884 ± 0.0048

Table S2. In vitro calibration measurements obtained from the calibration of the RgDAAO coated D-serine detecting MEA depicted in Figure 2.

**Parameters**	**Sensitivity**	**LOD**	**Linearity (R^2)**	**Selectivity against AA**
Measurements	9.32 pA/μM	0.16 μM	0.9997	263.82

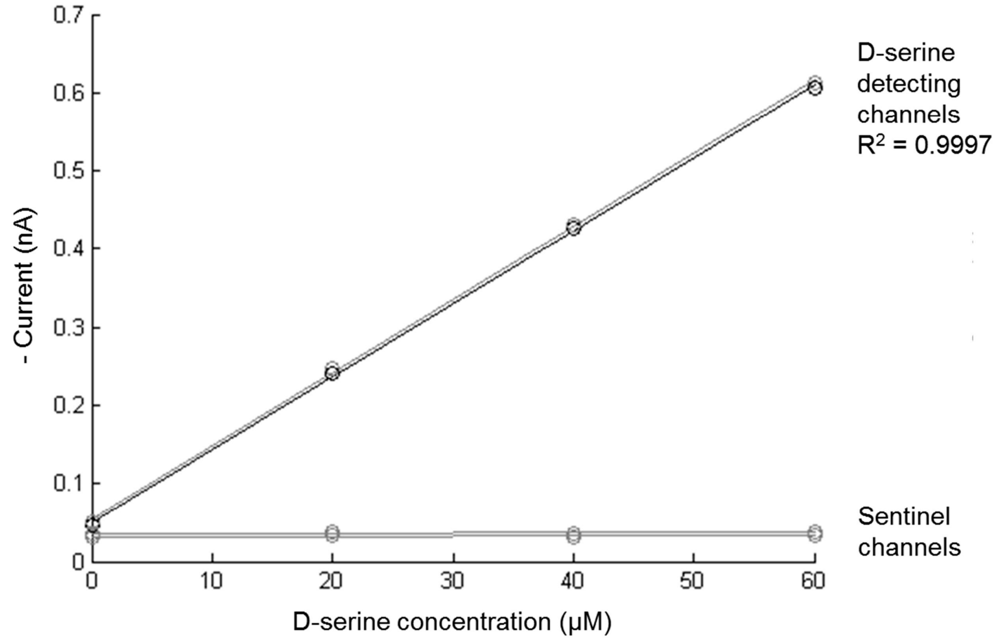

Figure S2. Linearity (R^2) curve obtained from the calibration of the RgDAAO coated D-serine detecting MEA depicted in Figure 2.
